# Decomposing Wealth-Based Inequalities in Neonatal Mortality in India: Evidence from National Family Health Survey (2019–2021)

**DOI:** 10.3390/ijerph23060795

**Published:** 2026-06-12

**Authors:** Diksha Gautam, Anuj Kumar Pandey, Benson Thomas M, Sutapa Bandyopadhyay Neogi

**Affiliations:** 1 SRM School of Public Health, Faculty of Medicine and Health Sciences, SRM Institute of Science and Technology, Kattankulathur, Chengalpattu 603203, Tamil Nadu, India; bensontm@srmist.edu.in; 2 Department of Health Systems and Implementation Research, International Institute of Health Management Research, New Delhi 110075, Delhi, India; anuj@iihmrdelhi.edu.in (A.K.P.); sutapa@iihmrdelhi.edu.in (S.B.N.); 3 Institute of Population and Social Research, Mahidol University, Nakhon Pathom 73170, Thailand; 4 Visiting Research Fellow, University of Huddersfield, Huddersfield HD1 3DH, UK

**Keywords:** neonatal mortality, wealth inequality, decomposition, concentration index

## Abstract

**Highlights:**

**Public health relevance—How does this work relate to a public health issue?**

- India contributes nearly one-fifth of global neonatal deaths, and it remains challenging to achieve the SDG 2030 target due to substantial wealth-based disparities.
- This study investigates how neonatal mortality is unequally distributed across socioeconomic groups and geographic contexts in India.

**Public health significance—Why is this work of significance to public health?**

- Neonatal mortality is disproportionately concentrated among the poorer, revealing persistent inequities despite the expansion of various national health programs focusing on disadvantaged populations.
- The observed inequality is substantially explained by modifiable factors, particularly household living conditions and maternal healthcare utilization.

**Public health implications—What are the key implications or messages for practi-tioners, policy makers and/or researchers in public health?**

- There is a need to strengthen the equity-oriented implementation of existing national health programs to improve reach among disadvantaged populations.
- Targeted, place-based strategies, including Aspirational Districts and Blocks Programmes, should be supported by strengthened monitoring of equity and quality of care.

**Abstract:**

India exhibits substantial variation in neonatal mortality across regions and socioeconomic groups. This study used nationally representative survey data (2019–2021) to examine wealth-based inequalities in neonatal mortality. Socioeconomic disparities were assessed using Erreygers’ Normalized Concentration Index (ECI) and concentration curves, with subgroup analyses by residence, state development status (Empowered Action Group (EAG) vs. non-EAG), district typology, and region. Inequality was further decomposed using the Wagstaff method. Analysis of 176,843 most recent live births revealed marked rural–urban disparities, with neonatal mortality in rural areas (18.3 per 1000 live births) 1.6 times higher than in urban areas (11.5). Neonatal mortality was significantly concentrated among poorer households (ECI: −0.0123; *p* < 0.001), with greater inequality in urban areas, EAG states, and non-aspirational districts. Regional variation was evident, with the highest inequality in the Western and Central regions. Decomposition analysis showed that inequality was primarily driven by adverse household conditions and maternal risk factors concentrated among poorer populations. Key contributors included unclean cooking fuel, higher parity, large family size, normal delivery and inadequate antenatal care. These findings highlight the need for equality-focused strategies addressing both social determinants and gaps in access to quality maternal and newborn care.

## 1. Background

Neonatal mortality, defined by the World Health Organization (WHO) as death within the first 28 days of life, constitutes a critical component of under-five mortality and is a key indicator of population health and health system performance [1]. Although substantial global reductions have been achieved and many countries are on track to meet the Sustainable Development Goal target of reducing neonatal mortality to 12 deaths per 1000 live births by 2030, neonatal deaths continue to account for nearly half of all under-five deaths globally, with the burden disproportionately concentrated in low- and middle-income countries [2].

India has achieved substantial reductions in neonatal mortality over the past decades, decreasing its contribution to the global burden from nearly one-third of neonatal deaths in 1990 to less than one-fifth in recent years [3]. Nevertheless, progress has slowed, with recent estimates indicating a stagnation of NMR at around 19 deaths per 1000 live births over the past three years [4]. With approximately 67,385 births occurring daily, an estimated 1440 neonatal deaths occur each day, representing a substantial and ongoing public health challenge [3]. However, national averages mask pronounced heterogeneity across geographic regions and socioeconomic groups. Significant disparities persist across states and districts, with northern and central states consistently reporting higher neonatal mortality compared to southern states [5]. While states such as Kerala, Tamil Nadu, Maharashtra, and Himachal Pradesh have already achieved the SDG target, others, including Madhya Pradesh, Uttar Pradesh, and Chhattisgarh, continue to report markedly higher NMR levels [4]. District-level evidence further indicates that only a small proportion of districts are likely to meet the SDG target, with a concentration of high-burden districts in socioeconomically disadvantaged regions, although pockets of high risk also exist within relatively better-performing states. Inter-state disparities in neonatal mortality in India narrowed between 2000 and 2019, primarily driven by reductions in inequality among high-mortality states [6]. A growing body of evidence indicates that neonatal mortality is closely linked to socioeconomic conditions at both individual and contextual levels [7]. Households in lower wealth quintiles, as well as those residing in rural and underserved areas, consistently experience higher risks of neonatal death compared to their wealthier and urban counterparts [5]. These disparities represent not only a major public health concern but also a preventable component of neonatal mortality.

In response, the Government of India (GoI) has introduced several maternal and newborn health initiatives, including the India Newborn Action Plan (INAP) and targeted place-based programs such as the Aspirational Districts and Aspirational Blocks Programmes aimed at improving maternal and child health outcomes in underserved regions [8,9,10,11]. Despite these efforts, substantial socioeconomic inequalities in neonatal mortality persist.

These approaches reflect an increasing emphasis on reducing geographic and socioeconomic inequalities. However, limited evidence exists on understanding the underlying determinants of wealth-based inequalities in neonatal mortality. Thus, the present study aims to examine wealth-based inequalities in neonatal mortality in India using nationally representative NFHS-5 data and to decompose the contribution of individual, household, community, health system, and newborn-level factors driving these disparities.

## 2. Methods

### 2.1. Study Design and Data Source

A secondary data analysis was carried out using data from the fifth round of the National Family Health Survey (NFHS-5), a nationally representative, cross-sectional household survey conducted during 2019–2021. NFHS-5 provides comprehensive information on demographic characteristics, maternal and child health, reproductive health, and healthcare utilization for 707 districts of 28 states and 8 union territories [12]. The data for this study were accessed from the Demographic and Health Surveys (DHS) Program data repository upon prior registration and approval [13]. The dataset is publicly available, fully anonymized, and does not contain any personally identifiable information.

Since the unit of analysis in the present study is children, the Birth Recode file (BR) has been used for the estimation of neonatal mortality. In addition to the BR file, relevant explanatory variables were drawn from the Individual Recode (IR) and Kids Recode (KR) files to ensure comprehensive coverage of maternal, household, and health system characteristics. These datasets were merged using unique identifiers provided in the NFHS-5 data structure to create a consolidated analytical dataset.

### 2.2. Study Population

Though the NFHS-5 data includes a total of 724,115 women aged 15–49 years with 257,995 births that occurred in the last five years, 4 preceding the survey, we have restricted the analysis to the 176,843 most recent live births to minimize recall bias and to ensure consistency in the measurement of maternal and health service-related variables.

### 2.3. Outcome Variable

The primary outcome of interest was neonatal mortality, defined as the death of a live-born infant within the first 28 completed days of life, in accordance with WHO definitions. A binary outcome variable was created, coded as 1 and 0, where “1” indicates that the child died within the neonatal period and “0” indicates that the child survived beyond 28 days.

### 2.4. Explanatory Variables

Explanatory variables were selected based on the existing literature and a conceptual framework capturing the multifactorial determinants of neonatal mortality. These variables were grouped into five domains to reflect different levels of influence.

Individual-level (maternal) factors included age of the mother at first birth (18–34 years, <18 and >34 years), parity (unavoidable first birth, up to 2 births, more than 2 births)—unavoidable first births were treated as a separate category because preceding birth interval-related risks are not applicable for primiparous births, maternal education (not educated, primary educated, secondary/higher educated), maternal height (>145 cms, ≤145 cms), high-risk fertility behavior (no, any), maternal substance use (tobacco and alcohol) (no, yes), pregnancy intention (yes, no more or later), experience of pregnancy-related complications (no, any one), and anemia status (non-anemic—Hb ≥ 11 g/dL, anemic—Hb < 11 g/dL). Household-level factors comprised wealth quintile (poorest, poorer, middle, richer, richest), source of drinking water (clean, unclean), type of cooking fuel (clean, unclean), sanitation facilities (clean, unclean), floor material (pakka, kaccha), exposure to mass media (less than/at least once a week, not at all), caste (General/other, OBC, ST, SC), religion (Hindu, Muslim/others), and household size (1–4, 5 and above). Community-level factors included place of residence (urban, rural), community wealth status (not poor, poor), community-level women’s education (high, low), geographic region (South, Central, North, East, Northeast, West), classification of states into Empowered Action Group (EAG) and non-EAG states, and district classification (aspirational, non-aspirational districts). Health system-related factors captured utilization and quality of maternal healthcare services, including timing of first antenatal care (ANC) visit (first trimester, second/third trimester), number of ANC visits (≥4, <4), knowledge of birth preparedness and complication readiness (BPCR) (knows about all components, none/some components), perceived quality of ANC (none/some, all), place of delivery (home, public facility, private facility), mode of delivery (other than cesarean, cesarean), and contact with frontline health workers (ASHA) (no, yes). Newborn care factors included early initiation of breastfeeding (more than one hour, within one hour), skin-to-skin contact (no, yes), and sex of the child (male, female).

Some variables mentioned above were created by calculating composite indexes using various dichotomous/nominal/ordinal variables:

- High-risk fertility behavior was defined as the presence of any of the following risk factors at the last childbirth: maternal age < 18 or ≥35 years, birth order ≥ 3, or birth interval ≤ 12 months. It is then categorized as no high-risk fertility behavior present, and at least one (any) present.
- Birth Preparedness and Complication Readiness (BPCR) was constructed using 11 indicators related to awareness of obstetric complications, institutional delivery, newborn care, breastfeeding, family planning, and emergency preparedness. The score ranged from 0–11 and was categorized as none/some and complete BPCR.
- Perceived quality of antenatal care was derived from five ANC service components: weight measurement, blood pressure examination, urine testing, blood testing, and iron supplementation. Scores ranged from 0–5 and were categorized as none/some and all services received.
- Experienced complications included convulsions, swelling, breech presentation, prolonged labour, and excessive bleeding, categorized as none, any one, any two, and three or more complications.
- Community-level variables, including community wealth status and women’s educational status, were generated by aggregating household wealth and women’s education indicators at the PSU level and categorized as high or low relative to the state average.

Missing data were assessed across all study variables, and those with less than 10% missingness were retained for analysis [14]. Multiple imputation by chained equations (MICE) [15] was employed under the assumption of missing at random, using relevant non-missing background characteristics, namely place of residence, wealth status and education [16,17,18]. Variables including maternal height, source of drinking water, type of cooking fuel, floor material, caste, timing and number of antenatal care (ANC) visits, type of toilet facility, maternal anemia status, and skin-to-skin contact were imputed.

### 2.5. Statistical Analysis

#### 2.5.1. Association of Determinants to Neonatal Mortality

The analysis was conducted using Stata version 17.0, accounting for the complex survey design through the application of sampling weights, clustering, and stratification using the svyset command. Descriptive statistics were used to estimate neonatal mortality rates (per 1000 live births) at the national and state levels, disaggregated by place of residence. Weighted percentages were calculated to describe the distribution of explanatory variables across the study population.

Bivariate associations between explanatory variables and neonatal mortality were assessed using binary logistic regression models. Results were presented as unadjusted odds ratios (ORs) with 95% confidence intervals (CIs).

To identify independent determinants of neonatal mortality, a sequential multivariable logistic regression approach was adopted using a hierarchical modelling framework. In Model I, all individual-level variables were entered simultaneously, and variables found to be statistically significant were retained for subsequent modelling. Model II incorporated the significant individual-level variables from Model I along with household-level variables. Model III included the significant variables retained from Model II together with community-level factors. In Model IV, significant variables from the previous model were combined with health-system-related variables. Finally, Model V included the significant variables retained from Model IV along with newborn care variables to obtain the final adjusted model. Adjusted odds ratios (AORs) with 95% confidence intervals were reported for the final model. Statistical significance was assessed at *p* < 0.05. A forest plot was generated based on the estimates from the final multivariable model to visually present the adjusted associations between explanatory variables and neonatal mortality.

#### 2.5.2. Measurement of Wealth-Based Inequality

The degree of wealth-based inequality in neonatal mortality was measured by using the concentration curve (CC). Even though CC gives a graphical view of the inequality, it failed to provide the numerical quantity of the magnitude of the inequality. Therefore, taking into consideration that the outcome variable (neonatal mortality) is binary, Erreygers’ normalized concentration index (ECI), which is suggested for such a rare condition, was used in our study to estimate the degree of inequality in neonatal mortality. The ECI corrects for the limitations of the standard concentration index when applied to dichotomous variables. The ECI asks, “How unequally is neonatal mortality distributed across socioeconomic rank, after correcting for its binary nature?”

The ECI can be expressed as:

$$ECI=\frac{8\;\times \mathrm{cov}\left(y_{i},r_{i}\right)}{b-a}$$

where ECI denotes the Erreygers’ concentration index, $r_{i}$ is the socioeconomic status ranking of individual i by wealth index, cov is covariance, and ‘b’ and ‘a’ represents the upper and lower bound of the outcome variable, respectively. The range (b−a) becomes one for a binary variable, as in our study. The ECI ranges between −1 and +1, with negative values indicating a disproportionate concentration of neonatal deaths among poorer households (pro-poor inequality), positive values indicating concentration among wealthier households (pro-rich inequality), and zero indicating no socioeconomic inequality [19].

The ECI was estimated at the national level and further stratified by place of residence, EAG classification, district typology (aspirational versus non-aspirational), and geographic regions to capture variations in inequality.

#### 2.5.3. Decomposition of Inequality

To quantify the contribution of different determinants to wealth-related inequality in neonatal mortality, a decomposition analysis of the ECI was performed using a regression-based approach. This method decomposes the overall inequality into the contributions of individual determinants based on their association with the outcome and their distribution across wealth groups.

The decomposition of ECI can be expressed as [20]:

$$ECI=4\left[\sum_{k}\beta {\bar{x}}_{k}C_{k}+GC_{\varepsilon }\right]$$

where βₖ represents the coefficient of determinant k, $\bar{x}ₖ$ is mean of determinant k, $C_{k}$ is the concentration index of the determinant, and GCε is the generalized concentration index for the error term ε. The first term represents the explained component of inequality, while the second term (GCε) captures the residual or unexplained inequality.

The contribution of each determinant to overall inequality was determined by two key components: (i) its elasticity (i.e., the sensitivity of neonatal mortality to that determinant), and (ii) the degree of socioeconomic inequality in that determinant. The percentage contribution of each factor was calculated by dividing its absolute contribution by the total concentration index and multiplying by 100. A negative contribution indicates that the determinant reduces inequality, whereas a positive contribution indicates that it increases inequality.

### 2.6. Ethical Consideration

The study is based on secondary analysis of publicly available, anonymized NFHS-5 data. Ethical approval for the survey was obtained by the original implementing agencies, and informed consent was secured from all participants prior to data collection. No additional ethical approval was required for this analysis.

## 3. Results

A total of 176,843 live births were included in the analysis. The overall NMR was 16.2 per 1000 live births, with rural NMR being 1.6 times (18.3 per 1000 live births) higher than in urban areas (11.5 per 1000 live births). Uttar Pradesh reported the highest NMR (25.9 per 1000 live births), followed by Bihar (23.2), Uttarakhand (21.6), and Jharkhand (21.1). The lowest NMRs were observed in Puducherry (1.1 per 1000 live births), Chandigarh (1.9), Kerala (2.9), Jammu and Kashmir (5.4), and Sikkim (5.6). In most states/UTs, neonatal mortality was higher in rural areas than in urban areas, with exceptions such as Odisha, Tripura, Mizoram, Nagaland, and Goa (Appendix A).

Table 1 presents the characteristics of individual, household, community, health system, and newborn care factors of the study population. The study population was predominantly composed of mothers aged 16–34 years, those with formal education, and women with parity of two births or fewer. High-risk fertility behavior and pregnancy-related complications were common among the study population. At the household level, most births belonged to households with improved drinking water, sanitation, and pucca housing conditions, while a considerable proportion were from poorer wealth quintiles and rural areas. Most births occurred in communities with relatively higher socioeconomic status and female educational attainment. Additionally, a large proportion of births were reported from EAG states and non-aspirational districts. Regarding health-system factors, most mothers initiated antenatal care during the first trimester, received four or more ANC visits, and delivered in public health facilities. In terms of newborn care, a large proportion of newborns received skin-to-skin contact and breastfeeding within one hour of birth.

### 3.1. Association of Determinants with Neonatal Mortality

**Figure 1:**
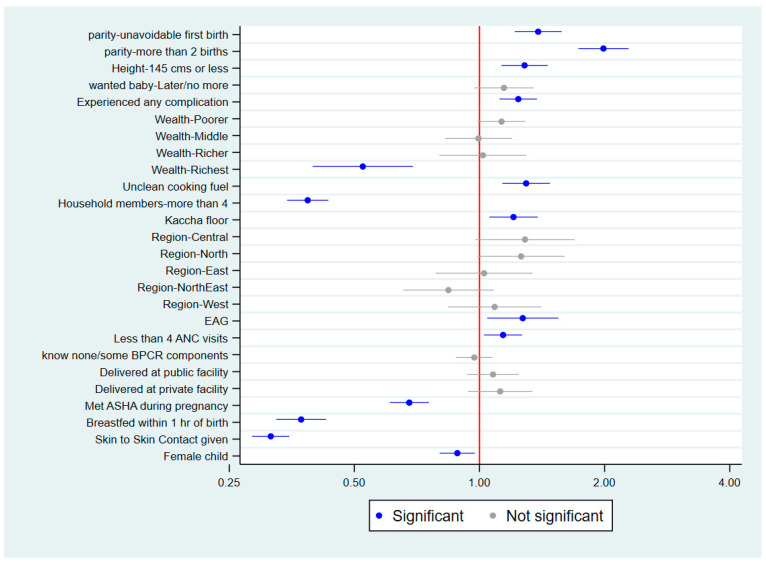
Adjusted odds ratios (AORs) and 95% confidence intervals for factors associated with neonatal mortality in India, NFHS-5 (2019–2021). Estimates are derived from the final hierarchical multivariable logistic regression model (Model V), with odds ratios adjusted for all variables retained in the final model. The vertical red line represents the null value (AOR = 1.0) indicating no association between the studies factors and neonatal mortality; estimates to the right of the line indicate increased odds, while those to the left indicate decreased odds.

The multivariable hierarchical logistic regression analysis identified several factors that remained independently associated with neonatal mortality after mutual adjustment for individual, household, community, health-system, and newborn-level factors (Appendix A). In the final adjusted model (Model V), neonates born to mothers with more than two births (AOR = 1.44; *p* < 0.001), short maternal stature (≤145 cm) (AOR = 1.28; *p* < 0.001), and pregnancy-related complications (AOR = 1.24; *p* < 0.001) exhibited significantly higher odds of neonatal mortality. Several household factors were independently associated with neonatal mortality, with significantly higher odds among households using unclean cooking fuel (AOR = 1.30; *p* < 0.001) and those living in kaccha houses (AOR = 1.21; *p* < 0.01). Compared with the poorest households, neonates from the richest households (AOR = 0.52; *p* < 0.001) and those with larger household size (AOR = 0.39; *p* < 0.001) had substantially lower odds of mortality. Regional disparities were also evident after adjustment, with neonates residing in EAG states experiencing significantly higher odds of neonatal mortality (AOR = 1.27; *p* < 0.05) (Figure 1).

Neonates born to mothers with fewer than four ANC visits had higher odds of mortality (AOR = 1.14; *p* < 0.05). Conversely, several health-system and newborn-care factors were associated with reduced odds of neonatal mortality, such as meeting ASHA workers during pregnancy (AOR = 0.68; *p* < 0.001), early initiation of breastfeeding within one hour of birth (AOR = 0.37; *p* < 0.001), and skin-to-skin contact (AOR = 0.31; *p* < 0.001). Female neonates also had lower odds of mortality compared with male neonates (AOR = 0.88; *p* < 0.05).

#### Magnitude of Wealth-Related Disparities in Neonatal Mortality

Table 2 presents the wealth-related inequalities in neonatal mortality. At the national level, wealth-related inequality in neonatal mortality was pro-poor, with an ECI of −0.0123 (*p* < 0.001), indicating a disproportionate concentration of neonatal deaths among poorer households.

Wealth inequality in neonatal mortality was more evident in urban (ECI: −0.0102; *p* < 0.001) as compared to rural areas (ECI: −0.0096; *p* < 0.001). By development classification, inequality was more pronounced in EAG states (ECI: −0.0100; *p* < 0.001) compared to non-EAG states (ECI: −0.0083; *p* < 0.001). Across district typologies, non-aspirational districts exhibited greater inequality (ECI: −0.0125; SE: 0.00074; *p* < 0.001) relative to aspirational districts (ECI: −0.0090; SE: 0.00167; *p* < 0.001).

**Figure 2:**
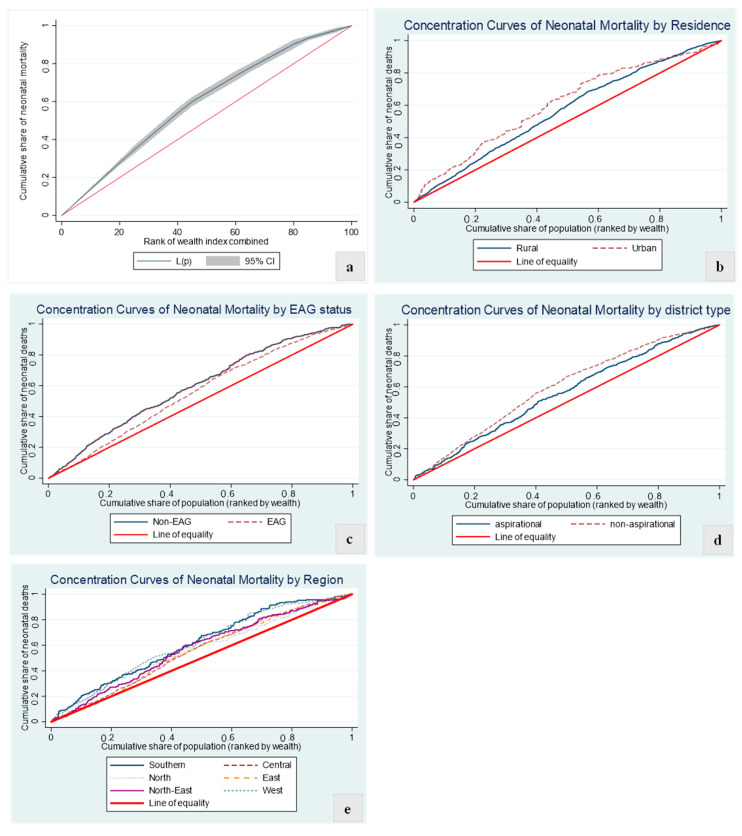
Concentration curve of wealth-related inequality in neonatal mortality in India (2019–2021): (**a**) national level; (**b**) by place of residence (rural and urban); (**c**) by EAG and non-EAG states; (**d**) by aspirational and non-aspirational; and (**e**) by region. The red bold line depicts line of inequality.

Substantial regional heterogeneity was also observed. Pro-poor inequality was more pronounced in the Western (ECI: −0.0108; *p* < 0.001) and Central regions (ECI: −0.0103; *p* < 0.001). This was followed by the North-Eastern (ECI: −0.0097; *p* < 0.001), Eastern (ECI: −0.0088; *p* < 0.001) and Southern regions (ECI: −0.0084; *p* < 0.001). The Northern region showed the lowest magnitude of inequality (ECI: −0.0064; *p*—0.0001). The concentration curves of neonatal mortality are presented in Figure 2a–e.

Considerable interstate heterogeneity in wealth-related inequality was observed. The highest pro-poor inequality was recorded in Uttarakhand (ECI: −0.0257; *p* < 0.001), followed by Chhattisgarh (−0.0173; *p* < 0.001), Himachal Pradesh (−0.0148; *p* = 0.008), Meghalaya (−0.0146; *p* < 0.001), Jharkhand (−0.0132; *p* < 0.001), and Odisha (−0.0128; *p* < 0.001). Significant pro-poor inequality was also observed in Maharashtra (−0.0126; *p* < 0.001), Uttar Pradesh (−0.0113; *p* < 0.001), Karnataka (−0.0112; *p* < 0.001), Gujarat (−0.0105; *p* < 0.001), and Assam (−0.0095; *p* = 0.001) (Appendix A).

### 3.2. Decomposition of Socioeconomic Inequality in Neonatal Mortality

Table 3 shows that most determinants exhibited negative ECIs at the individual, household and community levels, indicating that these factors were disproportionately concentrated among poorer households. In contrast, some health system and newborn-level factors, such as home delivery, normal vaginal delivery, no contact with ASHA during pregnancy, and no skin-to-skin contact, had positive ECIs, suggesting that these factors were concentrated among wealthy people. Few factors at the individual level, such as the presence of any high-risk fertility behavior and experience of complications during pregnancy, and at the household/community level, such as large family size and Muslim population, also present positive ECIs.

According to the decomposition analysis, wealth-related inequality in neonatal mortality was primarily explained by household environmental disadvantages and differential utilization of maternal and newborn healthcare services. Use of unclean cooking fuel (82.8%) emerged as the largest contributor to the observed pro-poor inequality in neonatal mortality, indicating its strong concentration among poorer households and its adverse association with neonatal survival. Other important contributors included high parity (>2 births) (26.8%), delayed initiation of breastfeeding (12.4%), larger household size (11.8%), normal vaginal delivery (11.2%), and inadequate antenatal care utilization (<4 ANC visits) (10.9%). Poor maternal education and delivery at a private facility further contributed to the concentration of neonatal mortality among poorer households. In contrast, factors such as lack of ASHA contact during pregnancy (−14.4%), home delivery (−10.9%), first birth (−8.3%), pregnancy complications (−3.5%), and no skin-to-skin contact (−3.1%) contributed in the opposite direction, thereby offsetting a portion of the observed inequality. Overall, the combined contribution of the included covariates exceeded the observed ECI by 28.97%, suggesting the presence of unexplained factors that operate in the opposite direction and partially offset the observed socioeconomic inequality.

## 4. Discussion

The present study provides comprehensive evidence on the magnitude and drivers of wealth-based inequalities in neonatal mortality in India using NFHS-5. Neonatal deaths remain disproportionately concentrated among poorer households across diverse geographic and developmental settings. The rural–urban disparities in neonatal mortality rate revealed by the study are consistent with estimates from the Sample Registration System (SRS) and previous empirical studies in India [4,21,22]. Evidence from successive rounds of the NFHS has consistently documented persistent socioeconomic inequalities in neonatal mortality in India. Although overall neonatal mortality has declined over time, improvements have not been equitably distributed across socioeconomic groups [23,24,25]. For instance, while the wealth gap in late neonatal mortality narrowed substantially from 12.0 to 2.9 deaths per 1000 live births between 1993 and 2021, the corresponding gap in early neonatal mortality remained virtually unchanged (18.3 to 18.4 deaths per 1000 live births) [25]. Similar patterns have been reported for infant mortality, where socioeconomic disparities continue to persist despite overall improvements in survival [26].

Wealth-related inequality was more pronounced in urban areas, EAG states and non-aspirational districts. Similar patterns of pro-poor inequality in neonatal mortality have been reported in other low- and middle-income countries, reinforcing the role of socioeconomic disadvantage as a key determinant of neonatal survival [7]. The greater inequality in urban areas aligns with emerging evidence on the “urban disadvantage paradox,” where urban averages often mask stark intra-urban inequalities, particularly among slum populations and informal settlements [27,28,29,30]. Studies have shown that urban poor populations often face barriers to accessing quality healthcare despite geographic proximity, including financial constraints, overcrowding, and fragmented service delivery systems [31,32,33]. The pronounced inequality in EAG states and non-aspirational districts further corroborates the existing literature [6,34]. These regions are characterized by weaker health systems, lower female literacy, higher fertility rates, and limited access to quality healthcare services, all of which contribute to higher neonatal mortality and greater inequality [35]. Studies have emphasized the need for context-specific health policy interventions tailored to the differential contributions of socioeconomic determinants to child health inequalities [30].

Interestingly, the study highlighted that wealth-related inequality was lower in aspirational districts compared with non-aspirational districts. This finding appears counterintuitive given the generally poorer health indicators of aspirational districts. One possible explanation is that aspirational districts are comparatively more homogeneously deprived, with elevated neonatal mortality distributed more uniformly across socioeconomic groups, thereby producing lower relative inequality despite a higher overall mortality burden. Alternatively, the lower relative inequality may partly reflect the targeted policy attention, intensified monitoring, and focused implementation strategies under the Aspirational Districts Programme [8]. However, further investigation is required to understand whether these patterns reflect actual improvements in equity or differences in the socioeconomic distribution of mortality within districts.

Substantial interstate variation in wealth-related inequality in neonatal mortality was also observed. States such as Uttarakhand, Chhattisgarh, Meghalaya, Jharkhand, and Odisha demonstrated particularly high wealth-related disparities in neonatal mortality. However, the estimates for certain smaller states and UTs should be interpreted cautiously because relatively smaller sample sizes may affect the precision and stability of ECI estimates. Although statistically significant inequalities were observed in several states, wider confidence intervals in smaller populations may limit comparability across regions. Additionally, the lower relative inequality observed in North India despite a high neonatal mortality burden may partly reflect the persistence of elevated mortality across multiple socioeconomic groups in these states. Previous evidence from India suggests that although wealth-based inequalities in child mortality have narrowed over time in several states, high-burden states continue to experience substantial overall mortality levels [24,30]. This indicates that reductions in relative inequality do not necessarily correspond to lower mortality burden.

Findings from the decomposition analysis demonstrated that wealth-related inequality in neonatal mortality was largely explained by the unequal distribution of adverse household conditions, maternal risk factors, and maternal healthcare utilization among poorer populations. Household-level factors, particularly the use of unclean cooking fuel, emerged as major contributors to the observed inequality, consistent with evidence linking indoor air pollution and inadequate living environments with adverse neonatal outcomes [36,37,38,39]. Maternal biological risk factors, such as higher parity and short stature, also contributed to inequality. These findings align with prior studies demonstrating that maternal nutritional status and reproductive patterns are strongly socially patterned and disproportionately concentrated among poorer populations [7].

An important contribution of this study is the identification of a dual pathway of inequality wherein poorer populations are simultaneously more exposed to adverse risk factors and less likely to access protective healthcare interventions. Risk-enhancing factors such as inadequate antenatal care utilization were concentrated among poorer households, whereas protective practices, including timely breastfeeding and improved maternal healthcare utilization, were more common among wealthier groups. Certain determinants, including home delivery, pregnancy complications, and lack of ASHA contact during pregnancy, demonstrated offsetting contributions. The offsetting contribution of a lack of ASHA contact may reflect the targeted engagement of ASHA workers with socioeconomically disadvantaged and high-risk women. Similarly, home delivery and pregnancy complications may reflect differences in healthcare-seeking behavior, referral patterns, and access to institutional care not fully captured in the survey data. The findings further suggest that improvements in service coverage alone may be insufficient to reduce inequality in neonatal mortality unless the quality and responsiveness of maternal healthcare services are simultaneously strengthened. Persistent socioeconomic disparities in education, autonomy, awareness, and financial capacity continue to influence the utilization of timely and quality maternal healthcare services among poorer women [23,26].

India has established a comprehensive policy and programmatic framework to improve maternal and newborn health across the continuum of care, including the National Health Mission, Janani Suraksha Yojana, Janani Shishu Suraksha Karyakram, Pradhan Mantri Surakshit Matritva Abhiyan, LaQshya, DAKSHATA, MusQan, SUMAN, etc., which collectively aim to strengthen care across the antenatal, intrapartum, and postnatal continuum. These programs have played an important role in expanding access to antenatal, delivery, and postnatal services and improving overall service coverage. However, the persistence of wealth-related disparities observed in the present study suggests that the benefits of these initiatives have not been equitably distributed. Socioeconomically disadvantaged populations continue to face barriers in accessing timely and quality care, suggesting that improvements in service provision have not been matched by effective reach and uptake among those most in need. These findings point to persistent challenges in ensuring that existing programs effectively reach and benefit the most disadvantaged populations.

### Strengths and Limitations

This study benefits from the use of nationally representative data, a large sample size, and robust analytical methods, including the use of the ECI and concentration curves. The ECI decomposition was adopted as it quantifies the contribution of determinants to socioeconomic inequality using the full wealth ranking of the population and accommodates the binary nature of neonatal mortality, unlike Kitagawa and Oaxaca–Blinder approaches that focus on differences between predefined groups [40,41]. Additionally, a key strength lies in the application of decomposition analysis using a comprehensive set of determinants spanning individual, household, community, health system, and newborn-level factors, which collectively accounted for more than 100% of the observed inequality. However, certain limitations must be acknowledged. The cross-sectional design precludes causal inference, and the reliance on self-reported data may introduce recall bias. Although restricting the analysis to the most recent birth reduces recall bias, it may introduce selection bias and may not fully capture the experience of all births occurring during the reference period. Additionally, unmeasured factors, such as quality of care at the facility level and neonatal clinical severity, may contribute to the unexplained component of inequality. The strong protective association observed for skin-to-skin contact should therefore be interpreted cautiously, as the NFHS dataset does not capture information on neonatal illness severity, raising the possibility of residual confounding and reverse causality. Further, a residual component remained, suggesting the influence of contextual, behavioral, and health-system factors not captured in the present analysis.

## 5. Conclusions and Implications

Neonatal mortality in India remains deeply shaped by broader socioeconomic and structural inequalities, extending beyond gaps in healthcare delivery. Reducing these disparities will require integrated strategies that address both the social determinants of health, including housing and energy access, and persistent health-system barriers related to the quality, continuity, and accessibility of care. Targeted interventions in high-burden regions, particularly EAG states and disadvantaged districts, should be complemented by within-area approaches that ensure the effective reach of the most socioeconomically vulnerable households. Programs such as the Aspirational Districts and Aspirational Blocks initiatives provide a valuable platform for addressing geographic disparities in neonatal survival. However, their effectiveness in reducing wealth-related inequalities should be systematically assessed and strengthened through targeted resource allocation and monitoring. Strengthening frontline health worker engagement, improving the quality of antenatal and delivery care, and promoting early newborn care practices are critical for reducing both mortality and inequality. While the present analysis identifies the populations and contexts in which disparities are concentrated, future qualitative and mixed-methods research is needed to elucidate the cultural, behavioral, and health-system factors that continue to constrain equitable utilization of maternal and newborn healthcare services among disadvantaged populations.

## Figures and Tables

**Table 1:** Characteristics of the individual, sociodemographic, healthcare, and newborn and PNC factors amongst recently delivered women having live birth in India (2019–2021).

Variables	Categories	Weighted Sample Live Births [N = 176,843]	Weighted Sample Live Births After Imputation [N = 176,843]	Neonatal Deaths	% of Neonatal Deaths
Individual-Level Factors					
Age of the mother at first birth (in years)	18–34	157,077	157,077	2517	1.6
	<18 & >34	19,766	19,766	353	1.8
Parity (no. of children)	Unavoidable first birth	59,620	59,620	986	1.6
	Upto 2 births	62,370	62,370	744	1.2
	More than 2 births	54,853	54,853	1139	2.1
High risk fertility behavior	No	5132	5132	105	2.1
	Any	171,711	171,711	2764	1.6
Education of mother	Educated	140,867	140,867	2048	1.4
	Not formally educated	35,976	35,976	821	2.3
Height of the mother (in cms)	>145	152,411	154,614	2337	1.5
	≤145	19,925	22,229	532	2.4
	Missing	4507			
Tobacco and alcohol consumption among mothers	No	163,847	163,847	2626	1.6
	Yes	12,996	12,996	243	1.9
Wanted pregnancy when became pregnant	Then	163,583	163,583	2581	1.6
	Later or no more	13,260	13,260	288	2.2
Experience of complications	No	62,078	62,078	919	1.5
	Any	114,765	114,765	1950	1.7
Anemia level	Non anemic	70,553	73,687	1108	1.5
	Anemic (<11 g/dL)	99,720	103,156	1761	1.7
	Missing	6570			
Household-level factors					
Wealth	Poorest	44,867	44,867	973	2.2
	Poorer	40,481	40,481	793	2.0
	Middle	34,569	34,569	491	1.4
	Richer	31,054	31,054	386	1.2
	Richest	25,872	25,872	226	0.9
Source of drinking water	Clean	137,072	140,549	2251	1.6
	Unclean	30,785	36,294	618	1.7
	Missing	8986			
Type of cooking fuel	Clean	79,150	82,459	998	1.2
	Unclean	89,182	94,384	1871	2.0
	Missing	8511			
Type of toilet	Clean	122,055	125,355	1751	1.4
	Unclean	45,876	51,488	1118	2.2
	Missing	8912			
Floor	Pakka	101,912	106,036	1368	1.3
	Kaccha	66,328	70,807	1501	2.1
	Missing	8603			
Exposure to media	Less than/at least once	127,846	127,846	1847	1.4
	Not at all	48,997	48,997	1022	2.1
Family size	1–4	49,945	49,945	1270	2.5
	5 and above	126,898	126,898	1599	1.3
Caste	General/Other	30,373	31,796	430	1.3
	OBC	67,024	68,310	1140	1.7
	ST	35,379	37,431	568	1.5
	SC	35,271	39,306	731	1.9
	Missing	8796			
Religion	Hindu	129,944	129,944	2249	1.7
	Muslim/others	46,899	46,899	620	1.3
Community-level factors					
Place of residence	Urban	37,975	37,975	444	1.2
	Rural	138,868	138,868	2425	1.7
Community wealth status	Not poor	127,324	127,324	1935	1.5
	Poor	49,519	49,519	934	1.9
Community women’s educational status	High	143,649	143,649	2138	1.5
	Low	33,194	33,194	731	2.2
Region	Southern	22,766	22,766	219	1.0
	Central	46,748	46,748	1068	2.3
	North	19,717	19,717	213	1.1
	Eastern	33,374	33,374	678	2.0
	Northeastern	27,460	27,460	327	1.2
	Western	26,778	26,778	364	1.4
EAG states	Non-EAG	90,784	90,784	1029	1.1
	EAG	86,059	86,059	1840	2.1
Aspirational districts	Aspirational	32,570	32,570	616	1.9
	Non-Aspirational	144,273	144,273	2253	1.6
Health-system factors					
Timing of first ANC	First trimester	123,817	125,594	1893	1.5
	2nd/3rd trimester	41,078	51,249	976	1.9
	Missing	11,948			
Number of ANC visits	≥4	101,435	102,360	1335	1.3
	<4	73,048	74,483	1534	2.1
	Missing	2360			
Told about BPCR	All component	104,996	104,996	1562	1.5
	None/some components	71,847	71,847	1307	1.8
Perceived quality of antenatal checkups	All	149,964	149,964	2237	1.5
	None/Some	26,879	26,879	632	2.3
Place of delivery	Home	21,219	21,219	461	2.2
	Public	114,952	114,952	1710	1.5
	Private	40,672	40,672	698	1.7
Mode of delivery	Other than caesarean section	139,084	139,084	2308	1.7
	Caesarean section	37,759	37,759	561	1.5
Met ASHA during pregnancy	No	101,164	101,164	1861	1.8
	Yes	75,679	75,679	1008	1.3
Newborn care factors					
Time of first breast feeding	More than 1 h	101,198	101,198	2368	2.3
	Within 1 h	75,645	75,645	501	0.7
Skin-to-skin contact	No	45,702	46,129	1504	3.3
	Yes	128,880	130,714	1365	1.0
	Missing	2261			
Sex of the child	Male	94,903	94,903	1674	1.8
	Female	81,940	81,940	1195	1.5

EAG: Empowered Action Group States; OBC: Other Backward Caste; SC: Schedule Caste; ST: Schedule Tribe; ANC: Antenatal Care; ASHA: Accredited Social Health Activist (trained female community health workers act as a bridge between marginalized rural/urban communities and the public healthcare system); BPCR: Birth Preparedness and Complication Readiness.

**Table 2:** Erreygers’ Normalized Concentration Index (ECI) of neonatal mortality in India at the national level, by place of residence, EAG/non-EAG states, type of districts, and region, using NFHS-5 (2019–2021).

Variable	Category	Live Births	ECI	Std. Error
National		176,843	−0.0123 ***	0.0007
Place of residence	Urban	37,975	−0.0102 ***	0.0012
	Rural	138,868	−0.0096 ***	0.0008
EAG/non-EAG	EAG	86,059	−0.0100 ***	0.0011
	Non-EAG	90,784	−0.0083 ***	0.0008
District type	Aspirational	32,570	−0.0090 ***	0.0017
	Non-aspirational	144,273	−0.0125 ***	0.0007
Region	Southern	22,766	−0.0084 ***	0.0014
	Central	46,748	−0.0103 ***	0.0016
	North	19,717	−0.0064 ***	0.0016
	Eastern	33,374	−0.0088 ***	0.0016
	Northeastern	27,460	−0.0097 ***	0.0016
	Western	26,778	−0.0108 ***	0.0015

Note: *p* value < 0.001—***; NFHS: National Family Health Survey; EAG: Empowered Action Group states.

**Table 3:** Decomposition of ECI for neonatal mortality in India,2019–2021.

	Determinants	Categories	Elasticity	ECI	Absolute	% Contribution
Individual level factors	Age at first birth	<18 & >34 years	−0.0010	−0.1177	0.0001	−0.9371
	Parity	Unavoidable first birth	0.0067	0.1529	0.0010	−8.2997
		More than 2 births	0.0131	−0.2525	−0.0033	26.7955
	High risk fertility behavior	Any	−0.0082	0.0258	−0.0002	1.7125
	Education of Mother	No education	0.0023	−0.3271	−0.0008	6.1646
		Primary	0.0009	−0.1204	−0.0001	0.9084
	Height of mother	≤145 cms	0.0025	−0.1219	−0.0003	2.4684
	Consumption of alcohol & tobacco by mother	Yes	0.0003	−0.0599	0.0000	0.1221
	Wanted pregnancy when became pregnant	Later or no	0.0007	−0.0348	0.0000	0.2085
	Experienced complications during pregnancy	Yes	0.0095	0.0455	0.0004	−3.4988
Household/community level factors	Type of cooking fuel	Unclean	0.0139	−0.7334	−0.0102	82.8439
	Exposure to media	Not at all	−0.0013	−0.4785	0.0006	−5.0468
	No. of members in the household	5 and more	−0.0436	0.0332	−0.0015	11.7623
	Caste	OBC	0.0031	0.0987	0.0003	−2.5188
		ST	0.0008	−0.1703	−0.0001	1.0534
		SC	0.0027	−0.1453	−0.0004	3.2035
	Religion	Muslim/others	−0.0004	0.0358	0.0000	0.1170
Health system and newborn care factors	Timing of 1st ANC	2nd/3rd trimester	−0.0011	−0.1985	0.0002	−1.7249
	No of ANC visits during pregnancy	less than 4	0.0053	−0.2555	−0.0014	10.9319
	Knowledge of bpcr components	none/some	−0.0014	−0.1005	0.0001	−1.1010
	Quality of ANC	none/some	0.0004	−0.1805	−0.0001	0.6359
	Place of delivery	private	0.0045	−0.2193	−0.0010	8.1023
		home	0.0036	0.3761	0.0014	−10.9858
	Mode of delivery	Normal	−0.0054	0.2537	−0.0014	11.2179
	Whether visited by/met ASHA during pregnancy	No	0.0122	0.1449	0.0018	−14.3753
	When child put to breast	More than one hour	0.0400	−0.0382	−0.0015	12.4205
	Skin-to-skin contact	No	0.0173	0.0224	0.0004	−3.1505
	Sex of the child	Female	−0.0037	−0.0025	0.0000	−0.0727
	Residual inequality				−0.0036	
	% Residual inequality				28.97%	

ECI: Erreygers’ Normalized Concentration Index; OBC: Other Backward Caste; SC: Schedule Caste; ST: Schedule Tribe; ANC: Antenatal Care; ASHA: Accredited Social Health Activist (trained female community health workers act as a bridge between marginalized rural/urban communities and the public healthcare system).

## Data Availability

The data used in this study are publicly available from the Demographic and Health Surveys (DHS) Program repository “https://dhsprogram.com/data/available-datasets.cfm (accessed on 20 March 2026)” upon registration and approval. This study utilized data from the National Family Health Survey (NFHS-5), India (2019–2021). No new data were generated for this study.

## Abbreviations

The following abbreviations are used in this manuscript:

CC	Concentration Curve
DHS	Demographic and Health Surveys
ECI	Erreygers’ Normalized Concentration Index
INAP	India Newborn Action Plan
MICE	Multiple Imputation by Chained Equations
NFHS	National Family Health Survey
NMR	Neonatal Mortality Rate
SDG	Sustainable Development Goals
WHO	World Health Organization

## Appendix A

**Table A1:** Neonatal Mortality Rate (per 1000 live births) for the nation and states: urban versus rural (NFHS-5).

State	Total Live Births	NMR	Live Births-Rural	NMR-Rural	Live Births-Urban	NMR-Urban
Uttar Pradesh	33,009.7	25.9	28,266.5	27.2	5455.9	21.4
Bihar	20,338.0	23.2	19,257.5	24.5	2079.3	14.5
Uttarakhand	1445.8	21.6	1060.2	23.5	362.8	17.6
Jharkhand	5238.0	21.1	4683.3	21.8	727.9	17.7
Dadra & Nagar Haveli	68.8	19.4	36.7	19.1	26.8	19.6
Madhya Pradesh	10,172.2	17.8	8396.5	18.4	1899.9	16.0
Chhattisgarh	3948.3	17.8	3401.1	19.9	638.6	10.3
Assam	5130.4	17.1	4963.5	17.8	451.0	12.3
Odisha	6145.4	16.7	5694.0	16.6	715.2	17.4
Gujarat	7621.8	14.6	5052.3	19.0	2286.0	7.9
Meghalaya	614.5	13.9	573.0	15.2	69.0	6.5
Andhra Pradesh	5467.8	13.9	4240.4	14.8	1211.2	11.5
Himachal Pradesh	799.9	13.8	768.2	15.3	74.3	3.2
Rajasthan	11,183.5	13.3	9617.6	13.9	1819.9	11.2
Telangana	3851.3	13.2	2580.8	14.7	1135.7	10.8
Tripura	550.5	12.5	458.2	11.9	100.2	14.7
Haryana	3338.8	12.2	2523.8	13.5	785.2	9.3
Punjab	3326.2	11.8	2314.4	13.9	921.4	8.1
NCT Of Delhi	2393.1	11.6	82.4	38.2	1764.7	10.7
West Bengal	13,897.0	10.8	11,113.5	11.2	2844.5	9.7
Ladakh	24.2	10.8	21.1	13.5	3.8	0.0
Manipur	371.1	10.4	267.4	13.9	96.4	3.7
Maharashtra	14,985.9	10.3	9076.5	11.7	5091.6	8.5
Karnataka	7859.8	9.5	5287.7	10.1	2303.4	8.4
Andaman & Nicobar Islands	35.5	9.0	20.7	2.1	12.7	16.9
Mizoram	141.7	8.1	75.3	5.7	55.5	10.5
Nagaland	159.4	7.7	124.9	7.0	34.4	9.4
Tamil Nadu	8743.3	7.1	5203.3	8.1	3034.8	5.9
Goa	177.6	6.5	76.9	0.0	81.7	10.8
Arunachal Pradesh	144.4	5.7	135.2	5.3	15.9	7.8
Sikkim	56.0	5.6	39.5	8.6	15.1	0.0
Jammu & Kashmir	1393.9	5.4	1155.5	6.1	256.9	3.4
Kerala	3957.4	2.9	2258.8	3.7	1440.7	2.0
Chandigarh	121.8	1.9	1.3	200.0	91.8	0.0
Puducherry	120.6	1.1	37.1	0.0	66.0	1.5
Lakshadweep	9.2	0.0	2.7	0.0	5.2	0.0
National	176,843	16.2	138,868	18.3	37,975	11.5

**Table A2:** Unadjusted and adjusted odds ratios from multivariable logistic regression analysis of determinants of neonatal mortality in India, NFHS-5 (2019–21).

	Variables	Categories	Unadjusted OR	Model I: Individual Level Factors	Model II: Individual + Household Level Factors	Model III: Individual + Household + Community Level Factors	Model IV: Individual + Household + Community + Health System Level Factors	Model V: Individual + Household + Community + Health System + Newborn Care Factors
Individual level factors	Age of the mother at first birth (in years)	18–34	Ref	Ref				
		<18 & >34	1.10 [0.98–1.23]	0.89 [0.76–1.05]				
	Parity (no. of children)	Unavoidable first birth	1.39 *** [1.26–1.53]	1.42 *** [1.25–1.62]	1.45 *** [1.28–1.65]	1.46 *** [1.29–1.66]	1.46 *** [1.28–1.65]	1.38 *** [1.22–1.58]
		upto 2 births	Ref	Ref	Ref	Ref	Ref	Ref
		more than 2 births	1.76 *** [1.60–1.93]	1.56 *** [1.37–1.77]	2.06 *** [1.79–2.37]	1.97 *** [1.71–2.26]	2.02 *** [1.71–2.27]	1.99 *** [1.73–2.28]
	High risk fertility behavior	No	Ref	Ref				
		Any	0.78 ** [0.64- 0.95]	0.88 [0.65–1.18]				
	Education of mother	Not educated	Ref	Ref	Ref	Ref		
		Primary	0.86 ** [0.76–0.97]	0.85 * [0.72–0.99]	0.88 [0.75–1.04]	0.92 [0.78–1.08]		
		Secondary or higher	0.59 *** [0.54–0.64]	0.63 *** [0.56–0.72]	0.79 *** [0.70–0.90]	0.87 * [0.76–0.99]		
	Height of the mother (in cms)	>145	Ref	Ref	Ref	Ref	Ref	Ref
		≤145	1.60 *** [1.45–1.76]	1.46 *** [1.29–1.66]	1.33 *** [1.18–1.52]	1.33 *** [1.17–1.51]	1.31 *** [1.15–1.49]	1.28 *** [1.13–1.46]
	Tobacco and alcohol consumption among mothers	No	Ref	Ref	Ref			
		Yes	1.17 ** [1.02–1.34]	1.25 * [1.03–1.51]	1.17 [0.97–1.43]			
	Wanted pregnancy when became pregnant	Then	Ref	Ref	Ref	Ref	Ref	Ref
		Later or no more	1.38 *** [1.22–1.57]	1.27 ** [1.08–1.50]	1.30 ** [1.10–1.53]	1.30 ** [1.10–1.53]	1.23 ** [1.05–1.46]	1.14 [0.97–1.35]
	Experience of complications	No	Ref	Ref	Ref	Ref	Ref	Ref
		Any	1.15 ** [1.06–1.24]	1.21 *** [1.09–1.33]	1.21 *** [1.09–1.34]	1.22 *** [1.10–1.35]	1.25 *** [1.13–1.38]	1.23 *** [1.12–1.37]
	Anemia level	Non anemic	Ref	Ref				
		Anemic	1.14 ** [1.05–1.23]	1.04 [0.94–1.15]				
Household level factors	Wealth	Poorest	Ref		Ref	Ref	Ref	Ref
		Poorer	0.9 ** [0.82–0.99]		1.31 *** [1.15–1.51]	1.24 ** [1.08–1.42]	1.18 ** [1.04–1.35]	1.13 [0.99–1.29]
		Middle	0.65 *** [0.58–0.72]		1.26 ** [1.04–1.54]	1.14 [0.93–1.29]	1.03 [0.85–1.24]	0.99 [0.82–1.19]
		Richer	0.57 *** [0.50–0.64]		1.44 ** [1.12–1.84]	1.26 [0.98–1.63]	1.04716405 [0.82–1.33]	1.2 [0.80–1.30]
		Richest	0.4 *** [0.34–0.46]		0.87 [0.66–1.16]	0.74 [0.54–1.00]	0.55 *** [0. 42–0.72]	0.52 *** [0.39–0.69]
	Source of drinking water	Clean source	Ref		Ref			
		Unclean source	1.06 [0.97–1.16]		0.96 [0.85–1.08]			
	Type of cooking fuel	Clean fuel	Ref		Ref	Ref	Ref	Ref
		Unclean fuel	1.65 *** [1.53–1.78]		1.41 *** [1.24–1.61]	1.27 *** [1.11–1.44]	1.31 *** [1.15–1.49]	1.29 *** [1.13–1.48]
	Exposure to media	Less than/at least once	Ref		Ref			
		Not at all	1.57 *** [1.45–1.69]		0.96 [0.86–1.08]			
	Type of toilet	Clean	Ref		Ref	Ref		
		Unclean	1.66 *** [1.54–1.78]		1.12 * [1.00–1.25]	1.07 [0.96–1.20]		
	Floor	Pakka	Ref		Ref	Ref	Ref	Ref
		kaccha	1.45 *** [1.34–1.57]		1.42 *** [1.24–1.62]	1.18 * [1.04–1.34]	1.19 ** [1.04–1.36]	1.20 * [1.06–1.38]
	Family size	1 to 4	Ref		Ref	Ref	Ref	Ref
		5-max	0.49 *** [0.45–0.53]		0.39 *** [0.35–0.44]	0.38 *** [0.34–0.42]	0.38 *** [0.34–0.43]	0.38 *** [0.34–0.43]
	Caste	General/Other	Ref		Ref			
		OBC	1.24 *** [1.11–1.38]		1.05 [0.91–1.23]			
		ST	1.12 [0.99–1.27]		0.95 [0.79–1.14]			
		SC	1.38 *** [1.23–1.56]		1.05 [0.89–1.24]			
	Religion	Hindu	Ref		Ref			
		Muslim/others	0.76 *** [0.70–0.83]		0.96 [0.84–1.09]			
Community level factors	Place of residence	Urban	Ref			Ref		
		Rural	1.50 *** [1.36–1.66]			1.05 [0.89–1.23]		
	Community women educational status	High	Ref			Ref		
		Low	1.24 *** [1.15–1.35]			1.06 [0.94–1.20]		
	Community wealth status	Not poor	Ref			Ref		
		Poor	1.49 *** [1.37–1.62]			1.02 [0.91–1.15]		
	Region	Southern	Ref			Ref	Ref	Ref
		Central	2.40 *** [2.07–2.79]			1.45 ** [1.10–1.90]	1.60 *** [1.22–2.11]	1.1.29 [0.98–1.69]
		North	1.12 [0.93–1.36]			1.39 ** [1.10–1.77]	1.49 *** [1.17–1.90]	1.25 [0.99–1.60]
		Eastern	2.13 *** [1.83–2.49]			1.10 [0.85–1.44]	1.11 [0.85–1.45]	1.02 [0.78–1.34]
		NorthEastern	1.24 ** [1.04–1.47]			1.18 [0.99–1.64]	1.29 * [1.00–1.66]	0.84 [0.65–1.08]
		Western	1.42 *** [1.20–1.68]			1.19 [0.92–1.55]	1.24 [0.96–1.61]	1.08 [0.84–1.40]
	EAG states	Non-EAG	Ref			Ref	Ref	Ref
		EAG	1.91 *** [1.76–2.06]			1.50 *** [1.24–1.81]	1.44 *** [1.18–1.74]	1.27 * [1.04–1.55]
	Aspirational districts	Aspirational	Ref			Ref		
		Non-Aspirational Districts	0.82 *** [0.75–0.90]			1.09 [0.97–1.23]		
Health system level factors	Timing of first ANC	First trimester	Ref				Ref	
		2nd/3rd Trimester	1.27 *** [1.17–1.37]				0.93 [0.83–1.05]	
	Number of ANC visits	Four or more	Ref				Ref	Ref
		Less than 4	1.59 *** [1.48–1.71]				1.21 *** [1.09–1.35]	1.14 [1.02–1.26]
	Know Birth Preparedness and Complication Readiness (BPCR)	All components	Ref				Ref	Ref
		None/Some	1.22 *** [1.14–1.32]				1.15 ** [1.04–1.28]	0.97 [0.88–1.07]
	Perceived quality of antenatal checkups	All	Ref				Ref	
		None/Some	1.59 *** [1.45–1.74]				1.11 [0.96–1.27]	
	Place of delivery	Home	Ref				Ref	Ref
		Public	0.68 *** [0.61–0.75]				0.91 [0.79–1.05]	1.08 [0.93–1.24]
		Private	0.79 *** [0.70–0.88]				1.22 * [0.58–0.73]	1.12 [0.94–1.34]
	Mode of delivery (C-section)	No	Ref				Ref	
		yes	0.89 ** [0.81–0.98]				1.14 [0.97–1.33]	
	Met ASHA during pregnancy	No	Ref				Ref	Ref
		yes	0.72 *** [0.67–0.78]				0.65 *** [0.58–0.73]	0.68 *** [0.61–0.75]
Newborn and PNC factors	Time of first breast feeding	More than 1 h	Ref					Ref
		within 1 h	0.28 *** [0.25–0.31]					0.37 *** [0.32–0.43]
	Skin-to-skin contact	no	Ref					Ref
		Yes	0.31 *** [0.29–0.34]					0.31 ** [0.28–0.35]
	Sex of the child	Male	Ref					Ref
		female	0.82 *** [0.76–0.89]					0.88 ** [0.80–0.97]

Note: *p* value < 0.001—***; *p* < 0.01—**; *p* < 0.05—*.; Grey area depicts factors not included in the multivariate analysis.

**Table A3:** State-wise Erreygers’ Normalized Concentration Index (ECI) of neonatal mortality in India (2019–2021).

States	Sample Size (N)	Index Value	Std. Error
Jammu & Kashmir	4898	0.00087381	0.002367
Himachal Pradesh	2145	−0.01480651 **	0.00560156
Punjab	4520	−0.00511727	0.00327864
Chandigarh	144	−0.00663842	0.0062258
Uttarakhand	2966	−0.02572491 ***	0.00594171
Haryana	5162	−0.00893712 **	0.00329249
Nct Of Delhi	2379	−0.00999317 *	0.00443417
Rajasthan	10,831	−0.00777192 **	0.00248994
Uttar Pradesh	25,556	−0.01128528 ***	0.00224121
Bihar	13,874	−0.00619495 *	0.00275042
Sikkim	569	−0.01102468	0.00685581
Arunachal Pradesh	4570	−0.00578821 *	0.00247349
Nagaland	2205	−0.00389755	0.0041274
Manipur	2511	0.00157851	0.00454108
Mizoram	1896	−0.00143167	0.00463498
Tripura	1860	−0.00539472	0.00570604
Meghalaya	4602	−0.01463162 ***	0.00374737
Assam	9247	−0.00952827 **	0.00293516
West Bengal	4894	−0.00360363	0.00327597
Jharkhand	7465	−0.01324217 ***	0.00356714
Odisha	7141	−0.01275591 ***	0.00338148
Chhattisgarh	6526	−0.01730091 ***	0.00367135
Madhya Pradesh	11,700	−0.0048374	0.00274633
Gujarat	7575	−0.01052558 ***	0.00310929
Dadra & Nagar Haveli & Daman & Diu	635	−0.02059293	0.01230344
Maharashtra	7415	−0.01256732 ***	0.00262703
Andhra Pradesh	2092	−0.00653947	0.00570927
Karnataka	6389	−0.01117162 ***	0.00271617
Goa	322	0.01096372	0.00919116
Kerala	2360	0.00130322	0.0023825
Tamil Nadu	5228	−0.00520028 *	0.0025745
Puducherry	616	−0.00117449	0.00284034
Andaman & Nicobar Islands	401	−0.00283609	0.01052417
Telangana	5429	−0.00870164 *	0.00346092
Ladakh	469	−0.00917274	0.01066802

Note: *p* value < 0.001—***; *p* <0.01—**; *p* <0.05—*.
